# A new clinical rating scale for work absence and productivity: validation in patients with major depressive disorder

**DOI:** 10.1186/1471-244X-9-78

**Published:** 2009-12-03

**Authors:** Raymond W Lam, Erin E Michalak, Lakshmi N Yatham

**Affiliations:** 1 Department of Psychiatry; University of BC; Mood Disorders Centre, UBC Hospital, Vancouver, Canada

## Abstract

**Background:**

The prevalence of major depressive disorder (MDD) is highest in working age people and depression causes significant impairment in occupational functioning. Work productivity and work absence should be incorporated into clinical assessments but currently available scales may not be optimized for clinical use. This study seeks to validate the Lam Employment Absence and Productivity Scale (LEAPS), a 10-item self-report questionnaire that takes 3-5 minutes to complete.

**Methods:**

The study sample consisted of consecutive patients attending a Mood Disorders outpatient clinic who were in full- or part-time paid work. All patients met DSM-IV criteria for MDD and completed during their intake assessment the LEAPS, the self-rated version of the Quick Inventory for Depressive Symptomatology (QIDS-SR), the Sheehan Disability Scale (SDS) and the Health and Work Performance Questionnaire (HPQ). Standard psychometric analyses for validation were conducted.

**Results:**

A total of 234 patients with MDD completed the assessments. The LEAPS displayed excellent internal consistency as assessed by Cronbach's alpha of 0.89. External validity was assessed by comparing the LEAPS to the other clinical and work functioning scales. The LEAPS total score was significantly correlated with the SDS work disability score (r = 0.63, p < 0.01) and the Global Work Performance rating from the HPQ (r = -0.79, p < 0.01). The LEAPS total score also increased with greater depression severity.

**Conclusion:**

The LEAPS displays good internal and external validity in a population of patients with MDD attending an outpatient clinic, which suggests that it may be a clinically useful tool to assess and monitor work functioning and productivity in depressed patients.

## Background

Mental illnesses in general, and major depressive disorder (MDD) in particular, are among the most common, disabling and costly of medical conditions. The total economic burden (both direct and indirect costs) of depression were estimated at over C$6 billion in Canada [1], US$83 billion in the United States [2], and €118 billion in Europe [3].

The prevalence of MDD in the general population is highest in those of typical working age (15-64 years) [4] and, given the nature of the physical and cognitive symptoms of depression, it is not surprising that the major portion of the economic burden of MDD arises from impairment in occupational functioning. Numerous studies have documented that clinical depression is associated with high rates of absenteeism, or time away from work. For example, depressed workers in the United States reported 1.5-3.2 more short-term work-disability days per month, compared to people who were not depressed [5], while a Canadian study found that approximately 2.5% of employees in 3 large companies had at least 1 depression-related short-term disability leave [6]. Similarly, in the European ESEMeD study, depressed workers had 3-4 times more work-loss days per month than those without depression [7].

While the economic costs of depression-related absenteeism are significant, they are dwarfed by those attributed to presenteeism, in which depressed workers stay at work but have reduced productivity as a result of their condition. In a community survey in Canada, 29% of people with a history of MDD in the past year reported reduced activities at work, compared to only 10% of people with no history of depression [8]. Almost half of people with chronic depression reported reduced productivity at work [9] and the costs of productivity losses associated with MDD have been estimated in the United States (in 2002) at over US$31 billion [10].

Given the magnitude of occupational impairment in MDD, it is important to include assessment of work functioning within the clinical evaluation and management of the condition. There are many validated scales used to measure work performance and productivity, including generic productivity scales (e.g., Work Limitations Questionnaire [11], Stanford Presenteeism Scale [12]) that are useful for comparisons with other disease conditions. However, there are few work performance scales designed specifically for use in a depressed population. A rationale for using disease-specific measures includes the potential for such scales to provide more specific information that might otherwise be missed or to be more sensitive to change than generic counterparts [13]. For example, a depression-specific scale for work functioning may prove useful as a clinical tool for monitoring progress during treatment and/or as an outcome measure in clinical trials for MDD. This study seeks to validate a new clinical rating scale for work functioning and productivity in patients with MDD.

## Methods

### Scale Development

The Lam Employment Absence and Productivity Scale (LEAPS) was designed to assess work functioning and impairment in a clinically depressed population. The items were constructed and selected based on a review of the literature on depressive symptoms and interference with work functioning, and on the common work-related problems experienced by people with depression.

The LEAPS (Additional file 1) is a self-rated questionnaire consisting of 10 items: the first item asks the respondent to list their occupation and the next two items ask about the number of work hours scheduled in the past two weeks and the number of work hours missed. These items assess absenteeism, which can be expressed as a proportion (%) of work hours scheduled. Finally, there are 7 items rated on a 5-point Likert scale with the following response format: 'None of the time (0%)', 'Some of the time (25%)', 'Half the time (50%)', 'Most of the time (75%)', 'All the time (100%)', scored as 0-4, respectively. The LEAPS total score therefore ranges from 0 to 28. A "productivity subscale" sums the scores from the 3 items assessing work functioning and productivity (doing less work, doing poor quality work, and making more mistakes).

### Subjects and Procedures

The validation sample consisted of consecutive patients with MDD attending a Mood Disorders clinic at a university teaching hospital. Patients were referred from primary care physicians and from community psychiatrists. Clinical assessments were conducted by board-certified psychiatrists. Diagnoses were assigned according to DSM-IV criteria based on clinical interviews supplemented by a symptom check list and all available medical information. Inclusion criteria for this study included a DSM-IV diagnosis of MDD; patients with bipolar disorder were excluded. Patients also had to be working, defined as paid work (employed or self-employed), either part-time or full-time. Patients on short-term or long-term work disability were excluded. This study was approved by the Clinical Research Ethics Board of the University of British Columbia.

Patients completed several questionnaires at initial assessment, including the Quick Inventory of Depressive Symptomatology, Self-Rated (QIDS-SR), a validated and widely used self-rated scale to assess severity and type of depressive symptoms [14]. In addition, subjects completed the Health and Work Performance Questionnaire (HPQ, [15]) and the Sheehan Disability Scale (SDS, [16]). The HPQ was developed for the World Health Organization as a depression-specific, self-rated questionnaire that assesses illness-related work absence (as number of hours/week), work productivity, Global Work Performance, and job-related accidents. The HPQ has been validated against objective measures of absence and performance in a number of blue-collar and white-collar occupations [17,18] and can be considered the "gold standard" productivity assessment. The SDS is a generic self-report inventory that assesses the degree to which symptoms have disrupted the person's work, social life, and family life. A single question assesses work/school impairment, formatted as 'The symptoms have disrupted your work/school work:' and rated on a 0-10 point scale ranging from 'Not at all (0)' through 'Mildly (1-3)', 'Moderately (4-6)' and 'Markedly (7-9)' to 'Extremely (10)'. There are two additional items which inquire about the number of days lost in the past month owing to absence or reduced productivity.

### Statistical Procedures

All results are reported as means ± standard deviations (SD). Construct validation of a scale for work functioning is complex because there are no definitive measures for the underlying construct. Hence, we conducted a series of scale validation procedures. *Internal consistency *(the degree to which the items of a scale measure the same construct) of the 7 LEAPS items was measured using Cronbach's alpha. To assess the structure of the LEAPS, a *factor analysis *was conducted using Principal Components Analysis with varimax rotation. *Convergent validity *is the degree of correlation between a new scale and previously validated measures of the same construct. This was assessed using two-tailed Pearson correlations between the LEAPS total score and scores on other scales measuring work productivity. In addition, work functioning would be expected to be more impaired as the depressive symptomatology worsens. Therefore, the LEAPS should *discriminate *between severity categories (e.g., minimally depressed versus more severely depressed) of depression. This was evaluated by examining mean scores on the LEAPS across the range of severity categories of the QIDS-SR, using one-way ANOVA. If the overall F was significant, post hoc pairwise comparisons between severity categories were examined using Tukey's HSD to control for multiple comparisons. All statistical analyses were conducted using SPSS, V.16 [19].

## Results

### Subject Demographic Variables

Table 1 shows the demographic and clinical information for the 234 subjects studied. The profile is typical of a mood disorders cohort attending a specialty clinic. The mean score on the QIDS-SR was 13.8 ± 5.9, indicating a moderate severity of depression. The subjects missed an average of 10 hours of work in the past 2 weeks owing to their symptoms, which represented 16% of the time they were scheduled to work.

**Table 1:** Demographic and clinical features of the validation sample (N = 234).

Variable	Mean ± SD
Age (years)	39.2 ± 11.7

Marital status (% of sample) (married/single/divorced/separated)	43/34/14/9

Number of episodes	2.5 ± 4.3

Duration of current episode (months)	6.9 ± 8.9

QIDS-SR score	13.8 ± 5.9

Number of hours in the past 2 weeks scheduled or expected to work	60.3 ± 22.4

Number of hours in the past 2 weeks missed from work	10.2 ± 17.8

% of work hours missed (per hours scheduled)	16.2% ± 27.0%

SD, standard deviation; QIDS-SR, Quick Inventory of Depressive Symptomatology, Self-Rated.

### Internal Consistency

The Cronbach's alpha for the 7 Likert-scored items on the LEAPS was 0.89, indicating that the LEAPS items showed high internal consistency.

### Factor Analysis

Table 2 shows the results of the factor analysis with varimax rotation conducted on the 7 Likert-scored items of the LEAPS. Two factors were identified on the Principal Components Analysis that accounted for 75% of the variance in the LEAPS total score. The first factor included the 3 items relating to work productivity, which accounted for 60% of the variance. The second factor comprised the 4 items relating to troublesome symptoms, which accounted for an additional 15% of the variance.

**Table 2:** Factor loadings of the 7 items on the LEAPS (Principal Components Analysis, using varimax rotation).

LEAPS Item	Factor 1 (Work productivity)	Factor 2 (Troublesome Symptoms)
Low energy or motivation	0.40	**0.72**

Poor concentration or memory	0.28	**0.78**

Anxiety or irritability	0.23	**0.82**

Getting less work done	**0.73**	0.46

Doing poor quality work	**0.85**	0.31

Making more mistakes	**0.90**	0.10

Having trouble getting along with people, or avoiding them	0.15	**0.86**

LEAPS, Lam Employment Absence and Productivity Scale.

### Convergent Validity

Table 3 shows the Pearson correlation matrix for the LEAPS total score and the productivity subscale score with other work functioning and productivity measures. There were significant correlations between the scores with all the other measures, including a high correlation with the "gold standard" HPQ Global Work Performance rating. Only a moderate correlation was found with the SDS Work score, likely explained by the fact the SDS score is comprised of a single item. The LEAPS total score and work productivity subscale score also explained more of the variance with '% hours of work missed' than either the SDS Work score (r = 0.24) or the HPQ Global Work Performance score (r = -0.37).

**Table 3:** Pearson correlations of LEAPS scores with other work functioning and productivity measures.

LEAPS score	SDS-Work	HPQ Global Work Performance	HPQ Productivity (4 items)	% of work hours missed in the past 2 weeks
Total Score*	0.63	-0.79	-0.70	0.41

Work productivity subscale (3 items) score*	0.50	-0.85	-0.77	0.45

LEAPS, Lam Employment Absence and Productivity Scale; SDS-Work, Sheehan Disability Scale, Work item; HPQ, Health and Work Performance Questionnaire.

*All correlations are significant at p < 0.01.

### Discrimination Between Depression Severity Categories

Table 4 shows the mean scores on the LEAPS for each of the severity categories of the QIDS-SR depressive symptom scale. There were significant differences in the LEAPS total scores overall (one-way ANOVA: F = 47.4, df = 4,229, p < 0.01). Post hoc Tukey's HSD tests showed significant differences (p < 0.05) between each pairwise comparison, except between the Severe and Very Severe categories. Similar results were seen with the LEAPS productivity subscale scores.

**Table 4:** Mean scores on the LEAPS total and Productivity Subscale versus depression severity (based on QIDS-SR score).

QIDS-SR Severity Category (score range)	N	LEAPS total score*	SD	LEAPS Productivity Subscale score*	SD
None to minimal (0-5)	25	2.6	2.3	0.6	0.9

Mild (6-10)	41	8.4	4.6	2.9	2.1

Moderate (11-15)	78	13.1	4.6	4.8	2.7

Severe (16-20)	57	15.7	5.7	6.6	3.2

Very Severe (21-27)	33	18.2	6.7	5.9	4.4

**Total**	**234**	**12.5**	**6.8**	**4.6**	**3.4**

QIDS-SR, Quick Inventory of Depressive Symptomatology, Self-Rated; LEAPS, Lam Employment Absence and Productivity Scale; SD, standard deviation.

* p < 0.05, one-way ANOVA using post hoc Tukey's Highly Significant Differences for all pairwise comparisons, except between Severe and Very Severe categories.

**Figure 1:**
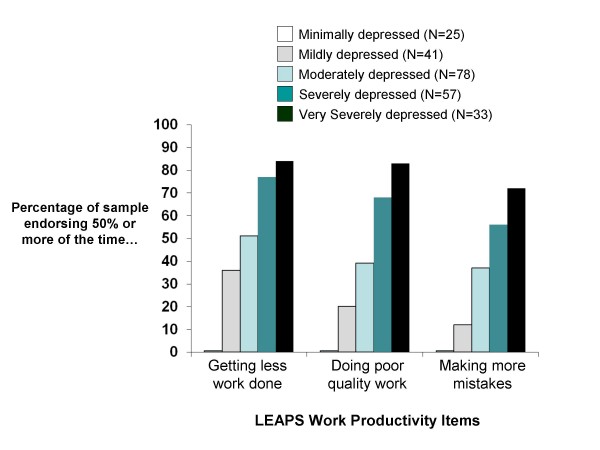
**Significant impairment in work productivity items (from the LEAPS) versus depression severity (based on QIDS-SR score)**.

Figure 1 shows the degree of clinical impairment (defined as percentage of the sample scoring 2 or higher on the item, indicating 50% or more of the time) in the individual productivity items associated with depression severity categories (as defined by the QIDS-SR scores).

## Discussion

The results from this validation study suggest that the psychometric properties of the LEAPS are very good. The LEAPS demonstrated a high internal consistency as measured by Cronbach's alpha. The factor analysis of the LEAPS showed that it is comprised of two factors, termed Work Productivity and Troublesome Symptoms, which account for a large proportion of the variance in total scores.

The validity of the LEAPS was further supported by the significant correlations with other validated measures of work functioning and productivity, including the SDS and the HPQ. Only a moderate correlation (explaining 40% of the variance) between the LEAPS and the SDS was observed, which is to be expected given that the SDS Work score is comprised of only a single item, compared to the higher correlation (explaining over 60% of the variance) found with the HPQ. The LEAPS score also showed higher correlations with the '% of work hours missed' over a 2-week period than the SDS Work score and the HPQ Global Work Performance rating.

The LEAPS scores also increase significantly with increasing overall severity of depressive symptoms and can discriminate between various depression severity categories, such as between 'None to minimal' and more severely depressed categories. The results from the individual productivity items on the LEAPS indicate that significant work impairment is found in patients with MDD. More than 75% of patients with higher severity of depressive symptoms described problems "much of the time" or "all the time" with the quantity and quality of work. In addition to productivity loss, the LEAPS data show that depressed patients were absent from work for 16% of their scheduled work hours (over 1.5 typical working days) in the previous two weeks. This is of similar magnitude to findings from other studies of work absence [5,7] and illustrates the substantial impact of depression on absenteeism.

Although the LEAPS performs well in this population, the limitations of this study need to be acknowledged. Further studies are needed to validate the LEAPS against external and objective measures of work performance, such as employer work absence data and objective measures of productivity. However, other studies have shown that self-rated work productivity measures are significantly correlated with objective metrics [20,21] and with administrative work records [15]. In addition, further studies are required to investigate the performance of the LEAPS in non-clinical samples of workers and in other clinical populations in specialist and primary care settings.

Clinical treatment studies in MDD now focus on symptom remission because of the evidence for poor outcomes predicted by the presence of residual depressive symptoms [22]. However, functional improvement, including that of work functioning, is more relevant to patients and restoration of occupational functioning is important to society [23]. The concept of measurement-based care for depression [24], in which outcomes are assessed using validated scales and which is increasingly recommended by clinical guidelines for the management of MDD [25], should encompass work functioning as well as symptom severity.

Many of the validated scales that assess work functioning are "generic" in that they are designed to evaluate productivity across a wide range of non-specific medical conditions. Alternatively, a disease-specific scale can provide important information for a defined clinical population. There are few depression-specific productivity scales available. The HPQ is the "gold standard" scale for assessment of work performance in patients with depression, but at 37 items and 8 pages in length, the respondent burden may be too high for routine clinical use. In contrast, the LEAPS is short (10 items on a single page) and simple and takes only 3-5 minutes to complete. Its brevity suggests that it will be an efficient tool for use in clinical settings. For example, the LEAPS can be used alongside symptom scales to monitor treatment progress, to ensure that work functioning improves in parallel with clinical symptoms. Additionally, scores on individual items (e.g., making mistakes) can be used to inform discussions with depressed workers regarding whether to stay at work or take time off while being treated for MDD.

The productivity impairment measured by the LEAPS increases, as expected, with increasing severity of depression. Although this is a cross-sectional observation, it suggests that the scale may also be useful as an outcome measure for occupational functioning in clinical trials of MDD. Further studies are underway to investigate the utility of the LEAPS to assess change in work functioning with treatment of MDD.

## Conclusion

The LEAPS is a short and simple self-rated scale of work absence and productivity that has been designed for use in a clinically depressed population. It displays good internal and external validity compared to other validated, self-rated scales of work performance and productivity. Further studies will be needed to determine whether the LEAPS can be used in other populations or as an outcome measure for clinical trials, and whether it will prove useful as a clinical tool to assess and monitor occupational functioning in patients with MDD.

## Competing interests

RWL has received honoraria for consulting/speaking from: Advanced Neuromodulation Systems Inc., AstraZeneca, Biovail, Canadian Network for Mood and Anxiety Treatments, Eli Lilly, Janssen, Litebook Company Ltd., Lundbeck, Lundbeck Institute, Servier, Takeda, and Wyeth. He has received research grants from: Advanced Neuromodulation Systems Inc., AstraZeneca, BrainCells Inc., Canadian Institutes of Health Research, Canadian Psychiatric Research Foundation, Litebook Company Ltd., Lundbeck, Mathematics of Information Technology and Advanced Computing Systems, Michael Smith Foundation for Health Research, Servier, and UBC Institute of Mental Health/Coast Capital Savings. He holds a copyright on the LEAPS.

EEM declares that she has no competing interests.

LNY has received honoraria for consulting/speaking from: AstraZeneca, Bristol Myers Squibb, Canadian Network for Mood and Anxiety Treatments, GlaxoSmithKline, Janssen, Pfizer, Ranbaxy, and Scherring Plough. He has received research grants from: AstraZeneca, Bristol Myers Squibb, Canadian Institutes of Health Research, Janssen, Michael Smith Foundation for Health Research, Servier, and Stanley Foundation.

## Authors' contributions

RWL conceived the study, designed the scale, contributed to data acquisition, conducted the statistical analysis, interpreted the data, wrote the initial draft of the manuscript, and funded the study through internal research funds. EEM contributed to study design and data acquisition, interpreted the data, and revised drafts of the manuscript. LNY contributed to study design and data acquisition, interpreted the data, and revised drafts of the manuscript. All authors read and approved the final manuscript.

## Pre-publication history

The pre-publication history for this paper can be accessed here:

http://www.biomedcentral.com/1471-244X/9/78/prepub

## Supplementary Material

Additional file 1 **The LEAPS**. The self-rated questionnaire consisting of 10 items that was used in the study. Click here for file
