# Supplementary material for: A new clinical rating scale for work absence and productivity: validation in patients with major depressive disorder
Source: BMC Psychiatry. 2009 Dec 3;9:78. doi: 10.1186/1471-244X-9-78 (PMC2793257; doi:10.1186/1471-244X-9-78)
Supplement: Additional file 1 — The LEAPS. The self-rated questionnaire consisting of 10 items that was used in the study. [file 1471-244X-9-78-S1.PDF]

Lam Employment Absence and Productivity Scale (LEAPS)

Name: ______________________________ Date: ____________________

Although all forms of work including house work, child care, and others are important, the next questions are about the employed or self-employed paid work that you may do. Please do not include house work, volunteer work, or school work.

1. What kind of paid work do you do? ________________________________

2. **Over the past 2 weeks**, how many hours were you scheduled or expected to work? __________

3. **Over the past 2 weeks**, how many hours of work did you miss because of the way you were feeling? __________

4. **Over the past 2 weeks,** how often at work were you bothered by any of the following problems? Please limit your answers to the time when you were at work. Please circle your ratings.

	**None of the time (0%)**	**Some of the time (25%)**	**Half the time (50%)**	**Most of the time (75%)**	**All of the time (100%)**
a) Low energy or motivation.	0	1	2	3	4
b) Poor concentration or memory.	0	1	2	3	4
c) Anxiety or irritability.	0	1	2	3	4
d) Getting less work done.	0	1	2	3	4
e) Doing poor quality work.	0	1	2	3	4
f) Making more mistakes.	0	1	2	3	4
g) Having trouble getting along with people, or avoiding them.	0	1	2	3	4
Add up score in each column:					

Total Score (0-28) = ________________

**Score**	**Work Impairment**
0-5	None to minimal
6-10	Mild
11-16	Moderate
17-22	Severe
23-28	Very severe

© Dr. Raymond W. Lam, 2009; permission for individual clinical use only, queries: r.lam@ubc.ca
